# Hypopharyngeal Rupture Following Indirect Neck Trauma due to a Car Accident in a 64-Year-Old Patient: A Case Report

**DOI:** 10.1155/carm/9933178

**Published:** 2025-07-26

**Authors:** Kiana Babaei, Ali Movahedi, Amirsadegh Alimardani

**Affiliations:** ^1^Department of Anesthesia, Neyshabur University of Medical Sciences, Neyshabur, Iran; ^2^Department of Basic Sciences, Neyshabur University of Medical Sciences, Neyshabur, Iran

**Keywords:** case report, hypopharyngeal rupture, indirect trauma

## Abstract

**Introduction:** Hypopharyngeal rupture caused by indirect neck trauma is a rare but potentially life-threatening injury. Delayed diagnosis can lead to severe complications, highlighting the importance of clinical suspicion and appropriate imaging.

**Case Presentation:** A 64-year-old male patient sustained indirect neck trauma following a car accident. He was initially transferred to the hospital with mild symptoms and was discharged. However, a few hours later, he returned to the emergency department with neck pain, odynophagia, and dysphagia. CT imaging revealed evidence of hypopharyngeal rupture accompanied by retropharyngeal emphysema. The patient was managed conservatively with Nil Per Os (NPO), intravenous antibiotics, and the placement of a nasogastric (NG) tube. He achieved full recovery without complications.

**Conclusion:** This case emphasizes the importance of thoroughly evaluating the relationship between clinical complaints and the mechanism of injury in patients with indirect neck trauma. High clinical suspicion, detailed history-taking, and appropriate imaging modalities are crucial for early diagnosis and effective management.

## 1. Introduction

The hypopharynx, also known as the laryngopharynx, is the lower part of the pharynx extending from the hyoid bone to the lower border of the cricoid cartilage. It consists of three regions: the piriform sinus, the posterior cricoid area, and the posterior pharyngeal wall [1]. Traumatic injuries in this region are classified into penetrating and nonpenetrating types. Although penetrating external injuries are rare, they can be life-threatening [2]. Nonpenetrating trauma, such as blunt force to the neck, may also lead to hypopharyngeal rupture [3]. Despite the rarity of such injuries, multiple case reports have been documented in the literature [4]. The delayed diagnosis of this injury, due to its uncommon nature and the potential lack of overt symptoms, may result in treatment delays, increased complications, and life-threatening outcomes [5].

Reported mechanisms contributing to hypopharyngeal rupture include sudden neck movements such as severe hyperextension or hyperflexion, sudden increases in intrapharyngeal pressure, or indirect compressive forces sustained during motor vehicle accidents. The diagnosis is challenging due to the broad spectrum of symptoms, ranging from mild dysphagia and hemoptysis to severe respiratory distress and subcutaneous emphysema [4–6]. Accurate diagnosis requires a thorough clinical evaluation and imaging modalities such as CT and MRI [7]. In addition, direct laryngoscopy plays a significant role in precisely identifying the rupture site [4–8].

Given the rarity of this injury and the absence of clear clinical signs, early diagnosis and timely intervention are crucial for patient prognosis and recovery. Recognizing the relationship between clinical complaints and the mechanism of injury is a key factor in preventing diagnostic delays and associated complications. Therefore, reporting rare and unusual cases not only enhances clinicians' awareness but also contributes to timely diagnosis and effective management strategies. In this study, we present a case of hypopharyngeal rupture caused by indirect neck trauma in a 64-year-old patient.

## 2. Case Presentation

**Figure 1:**
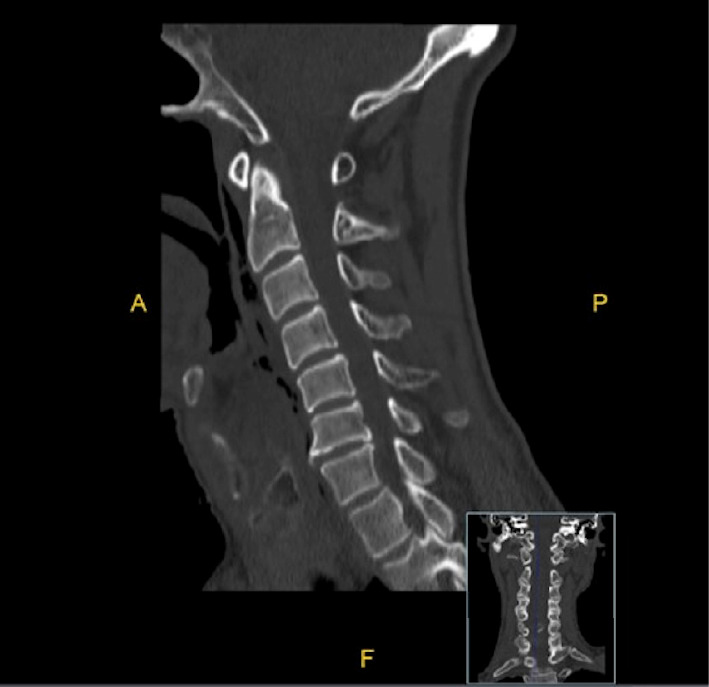
Sagittal CT scan of the soft tissue of the neck showing a longitudinal tear in the posterior esophagus from the beginning of the hypopharynx to the C5 vertebra.

**Figure 2:**
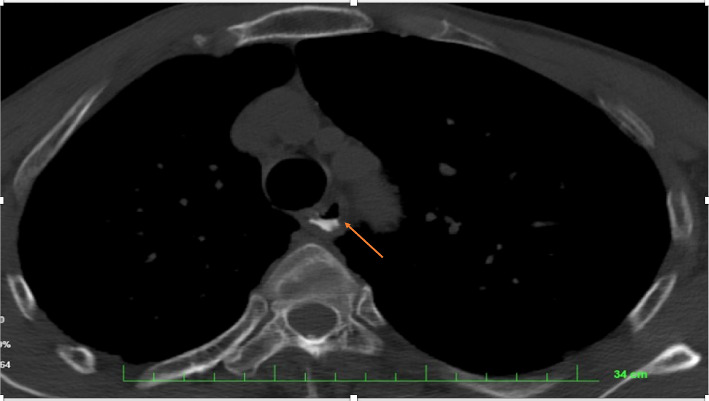
Leakage of contrast medium into the extra-luminal space, suggesting esophageal perforation (extravasation air).

A 64-year-old male patient, with no known underlying medical conditions, was a three-wheeler driver who sustained trauma after colliding with a parked Nissan vehicle. He presented with a left eyelid laceration, tenderness in the right lower leg and ankle, and mild neck pain. The initial evaluation included assessment of vital signs, bleeding control, and wound dressing. The patient was fully conscious and was transferred to the hospital under continuous monitoring by emergency medical personnel. He was examined by a general practitioner in the emergency department and subsequently discharged. Eight hours after discharge, the patient returned to the emergency department complaining of anterior neck pain, dysphagia, and odynophagia. On initial evaluation, he was hemodynamically stable with the following vital signs: GCS: 15, SPO_2_: 95%, BP: 130/80 mmHg, HR: 78 bpm, T: 36.2°C, BS: 152 mg/dL, and RR: 19. At the time of re-evaluation by the emergency medicine specialist, the patient had an oxygen saturation (SpO_2_) of 91% and a respiratory rate of 24 breaths per minute. Based on these parameters, oxygen therapy via nasal cannula was initiated. Physical examination by an emergency medicine specialist revealed mild swelling in the neck, with no signs of subcutaneous emphysema and dysphonia. Diagnostic and therapeutic measures, including laboratory tests, cardiac monitoring, urinary catheterization, oxygen therapy, and medication administration, were initiated. The patient received intravenous clindamycin (900 mg), ceftriaxone (1 g), and metronidazole (600 mg). Imaging studies included a CT scan of the brain and soft tissue of the neck. The cervical spine CT revealed degenerative changes (DJD) but showed no evidence of displacement or obvious fractures. Spiral CT with gastrografin contrast demonstrated irregularities in the proximal esophageal wall at the level of C1 and C2 vertebrae (Figure 1). In addition, gas densities were noted in the retropharyngeal and paravertebral spaces, along with a minor leakage of contrast medium into the extra-luminal space, suggesting esophageal perforation (Figure 2). No pleural or pericardial effusion or pneumothorax was detected on CT evaluation. Although the CT scan described changes at the C1–C2 levels, these correspond to the anatomical location of the hypopharynx, consistent with the findings from direct laryngoscopy. Following these findings, the patient was referred to general surgery and ENT specialists for further assessment. In the ENT consultation, the patient was kept Nil Per Os (NPO), and a revised medication regimen was prescribed, including intravenous clindamycin (600 mg), ceftriaxone (1 g), pantoprazole (40 mg), dextrose-saline infusion, and intramuscular pethidine (12.5 mg as needed). It was decided to proceed with direct laryngoscopy in the operating room. Under general anesthesia, direct laryngoscopy revealed a 2.5-cm perforation in the posterior wall of the hypopharynx, while the remaining laryngeal structures appeared normal. Following confirmation of the diagnosis, an NG tube was placed under laryngoscopic guidance. The patient's nutritional regimen consisted of 50-mL gavage feeding every 3 h, which was gradually increased to 150 mL every 3 h based on tolerance. The patient remained on NG tube feeding for 15 days, with instructions for an outpatient follow-up evaluation before tube removal. After 5 days of hospitalization, the patient was discharged in stable general condition with relative clinical improvement. In addition, in conjunction with ENT and general surgery consultations, ophthalmology and neurosurgery specialists assessed the patient to rule out any associated injuries. The patient underwent two follow-up visits: the first at 15 days and the second at 1 month postdischarge. On both occasions, he was evaluated at the ENT clinic and was found to be in good general condition with no complaints. Given his stable clinical status, tolerance of oral feeding, and absence of fever, dysphagia, or pain, the NG tube—placed initially—was removed at the 15-day visit.

## 3. Discussion

The present study reports a rare case of hypopharyngeal rupture due to indirect neck trauma in a 64-year-old patient. This injury was diagnosed by an Emergency Medicine Specialist following a motor vehicle accident and the patient's subsequent secondary hospital visit. During the initial evaluation by a general practitioner in the emergency department, the patient's complaint of mild neck pain was overlooked, and he was discharged. Upon representation, a detailed history-taking by the emergency medicine specialist and the correlation between the patient's clinical complaints (odynophagia) and the mechanism of injury (motor vehicle accident) raised clinical suspicion of hypopharyngeal rupture, leading to targeted diagnostic measures. Our findings are consistent with previous studies indicating that hypopharyngeal injuries following blunt trauma are rare but potentially life-threatening [3, 4]. According to past studies, hypopharyngeal rupture accounts for less than 2% of all upper aerodigestive tract perforations [9]. In addition, the most commonly reported cause of these injuries is motor vehicle accidents, including those involving cars, motorcycles, and bicycles [7]. Other proposed mechanisms in previous research include sudden neck movements, increased intrapharyngeal pressure, and indirect compressive forces [4, 6]. Killian's Dehiscence, an area of structural weakness at the junction of the hypopharynx and esophagus adjacent to the cricopharyngeus muscle, consists only of mucosa and serosa, making it particularly susceptible to rupture [10, 11]. In this case, indirect compressive force from the accident led to rupture in this region.

The clinical manifestations of hypopharyngeal rupture vary widely. Common symptoms include subcutaneous emphysema, stridor, hoarseness, dysphagia, and chest or neck pain [4, 9, 12]. In delayed cases, sore throat, fever, and neck swelling may indicate the development of a retropharyngeal abscess [4]. Our patient presented with neck pain and dysphagia, highlighting the importance of a thorough evaluation of the trauma mechanism and post-trauma follow-up. The symptoms of this injury can be nonspecific, leading to a delayed diagnosis [5]. Therefore, early recognition is crucial to preventing severe complications such as mediastinitis, fistula formation, and infectious abscesses [6]. Given the high morbidity and mortality associated with this injury, maintaining a high index of suspicion and conducting a meticulous patient assessment are the key to early diagnosis [2, 4]. In our case, despite the patient's mild pain during the initial visit, this symptom was not given adequate consideration by the general practitioner in the emergency department, resulting in premature discharge.

Due to the rarity of these injuries and the lack of standardized management guidelines for pharyngeal and esophageal ruptures, timely diagnosis is essential [6]. There is no consensus on the best diagnostic approach for this type of injury. Some studies suggest a combination of plain radiography, computed tomography (CT), fluoroscopy, and nasopharyngolaryngoscopy for evaluation [2, 6, 13]. The differential diagnosis includes retropharyngeal abscess, atlanto-occipital joint dislocation, and pneumomediastinum [2]. In addition, the presence of mediastinal, pericardial, or subcutaneous emphysema on plain chest radiography should raise suspicion for pharyngeal rupture [9]. In our patient, neck CT and spiral CT esophagography were performed to confirm the diagnosis. CT scanning can reveal the presence of air in fascial spaces and assist in surgical planning [8, 9]. Moreover, endoscopy plays a crucial role in confirming the presence, location, and extent of the rupture [14]. In our patient, direct laryngoscopy under general anesthesia was performed, revealing a 2.5-cm rupture in the posterior wall of the hypopharynx.

Although literature suggests surgical management for defects larger than 2 cm, our patient's stable hemodynamic condition, absence of systemic infection signs, and favorable response to conservative treatment led to a multidisciplinary decision to proceed nonoperatively. This reflects the importance of individualized patient management based on the clinical status. The management of hypopharyngeal rupture depends on factors such as the size and location of the injury, the patient's hemodynamic status, and the presence of associated complications [15]. Studies have shown that in hemodynamically stable patients with perforations smaller than 2 cm, conservative management—including NPO, broad-spectrum antibiotic therapy, and parenteral nutrition—can be effective [15]. Smith's study reported that hypopharyngeal perforations smaller than 2 cm could be managed conservatively whereas larger perforations or those involving the esophagus require surgical intervention [4]. In our case, the treatment approach included direct laryngoscopy and the placement of a nasogastric (NG) tube. This method was aimed at facilitating rupture healing while ensuring adequate nutritional support. The patient remained on NG feeding for 15 days and was closely monitored. Recognizing patient symptoms, obtaining a thorough history, considering the trauma mechanism, maintaining a high suspicion for hypopharyngeal rupture, conducting targeted diagnostic evaluations, selecting appropriate imaging modalities, ensuring timely follow-up, and implementing proper therapeutic interventions played a crucial role in the successful management of this patient.

## 4. Conclusion

Hypopharyngeal rupture due to indirect trauma is a rare but potentially life-threatening injury, with a challenging diagnosis due to its nonspecific symptoms and the possibility of delayed presentation. The findings of this study emphasize that maintaining a high index of suspicion, using appropriate imaging techniques, and conducting a thorough evaluation of patients with neck trauma—even in cases with initially mild symptoms—are essential for reducing complications and improving prognosis. Recognizing the correlation between clinical complaints and the mechanism of injury is the key to identifying these injuries and preventing diagnostic delays.

Reporting such rare cases can contribute to the advancement of clinical knowledge and the development of more effective management protocols. Given that emergency medicine is a specialized field and that the current shortage of emergency medicine specialists in Iran limits comprehensive coverage across all hospitals, the importance of training general practitioners working in emergency departments becomes even more significant. Increasing their awareness of the relationship between clinical complaints and injury mechanisms could play a vital role in improving the diagnostic and management process for these patients.

## Data Availability

The data that support the findings of this study are available from the corresponding author upon reasonable request.
